# Development of Combining of Human Bronchial Mucosa Models with Xpose*ALI*® for Exposure of Air Pollution Nanoparticles

**DOI:** 10.1371/journal.pone.0170428

**Published:** 2017-01-20

**Authors:** Jie Ji, Anna Hedelin, Maria Malmlöf, Vadim Kessler, Gulaim Seisenbaeva, Per Gerde, Lena Palmberg

**Affiliations:** 1 Lung and Airway Research, Institute of Environmental Medicine, Karolinska Institutet, Stockholm, Sweden; 2 Inhalation Sciences Sweden AB, Stockholm, Sweden; 3 Experimental Asthma and Allergy Research, Institute of Environmental Medicine, Karolinska Institutet, Stockholm, Sweden; 4 Inorganic Bionanotechnology Unit, Department of Chemistry and Biotechnology, Swedish University of Agricultural Sciences (SLU), Uppsala, Sweden; VIT University, INDIA

## Abstract

**Background:**

Exposure to agents via inhalation is of great concerns both in workplace environment and in the daily contact with particles in the ambient air. Reliable human airway exposure systems will most likely replace animal experiment in future toxicity assessment studies of inhaled agents.

**Methods:**

In this study, we successfully established a combination of an exposure system (Xpose*ALI*) with 3D models mimicking both healthy and chronic bronchitis-like mucosa by co-culturing human primary bronchial epithelial cells (PBEC) and fibroblast at air-liquid interface (ALI). Light-, confocal microscopy, scanning- and transmission electron microscopy, transepithelial electrical resistance (TEER) measurement and RT-PCR were performed to identify how the PBEC differentiated under ALI culture condition. Both models were exposed to palladium (Pd) nanoparticles which sized 6–10 nm, analogous to those released from modern car catalysts, at three different concentrations utilizing the Xpose*ALI* module of the PreciseInhale^®^ exposure system.

**Results:**

Exposing the 3D models to Pd nanoparticles induced increased secretion of IL-8, yet the chronic bronchitis-like model released significantly more IL-8 than the normal model. The levels of IL-8 in basal medium (BM) and apical lavage medium (AM) were in the same ranges, but the secretion of MMP-9 was significantly higher in the AM compared to the BM.

**Conclusion:**

This combination of relevant human bronchial mucosa models and sophisticated exposure system can mimic *in vivo* conditions and serve as a useful alternative animal testing tool when studying adverse effects in humans exposed to aerosols, air pollutants or particles in an occupational setting.

## Introduction

As more and more ethical concerns are raised over the use of animal models in medical research, attempts are made to reduce and replace animal experiments. For primary contact organs like the lung, *in vivo* exposure occurs at an air-liquid interface (ALI) close to the apical cell surfaces while *in vitro* submerge exposure poorly represents the *in vivo* route of exposure. Moreover, when exposing epithelial cells *in vitro* to particles under submerged condition, a substantial fraction of the particles will either remain in the liquid or be lost to the lateral walls of the culture vessel, which alter the dose of particles as well as the interaction with the cells. Some *in vitro* techniques for exposing primary or cell line cultures in ALI have been described in the literature[1–3], also several ALI cell cultures are commercially available and have been used for inhalation toxicology research, for instance MucilAir^TM^-HF (Epithelix, Genève, Switzerland) and EpiAirway^TM^ (MatTek, Ashland, USA).

Chronic exposure to particulate matter (PM) from traffic emissions or cigarette smoke is associated with higher risk of morbidity and mortality related to cardiovascular diseases, cancer and pulmonary diseases including chronic obstructive pulmonary disease (COPD) with or without chronic bronchitis [4, 5]. COPD affects up to 10% of the population world-wide and is estimated to become the third most common cause of disease-related death in 2020 [6, 7]. The disease is characterized by inflammation of the large airways (bronchitis), small airways (bronchiolitis) and destruction of the pulmonary parenchyma with formation of large airway spaces (emphysema). Chronic bronchitis is a commonly neglected co-morbidity of COPD that further increases morbidity and mortality in an independent manner [8]. Acute exacerbations of COPD and chronic bronchitis have been associated with short-term exposure to air pollution [9]. Therefore, using normal and chronic bronchitis-like models would give better insight regarding effects of particle exposure in health and disease. According to previous studies, treatment of epithelial cells with Interleukin-13 (IL-13) induced mucus-producing cells, metaplasia or hyperplasia as well as increased expression and production of MUC5AC[10]. These features are major characteristics of chronic bronchitis. Therefore, treating cell cultured under ALI conditions with IL-13, the development of a chronic bronchitis-like mucosa is expected. Xpose*ALI* is an *in vitro* cell exposure system where cells cultivated under ALI conditions can be exposed to aerosol of interest by being coupled to the PreciseInhale aerosol generator which generates aerosols from a variety of dry powders through de-agglomeration [11]. This set-up allows aerosol exposures of cell cultures to respirable particles. In addition, it only consumes minimal amounts of test substance which is favorable if the quantities are limited [12]. A variety of aerosols, including PM can be generated and precisely dosed to expose cells under well-controlled conditions.

In this methodological study, our primary goal was to develop a system which combined normal and chronic bronchitis-like models of bronchial mucosa, cultured at ALI with the newly developed Xpose*ALI* exposure module. These normal and chronic bronchitis-like models were exposed to aerosols of Pd nanoparticles, which are present in air pollution.

## Materials and Methods

### Establishment of bronchial mucosa models

The bronchial mucosa model (3D model) was generated by co-culturing primary bronchial epithelial cells (PBEC) from 3 different donors (All donors gave their informed and written consent and the study was approved by the Ethics Committee of Karolinska Institutet, Stockholm, Sweden) and MRC-5 cell (human fetal lung fibroblast cell line (American Type Culture Collection ATCC, Manassas, VA)) serving both as stromal cells and a feeding layer (S1 Information), on 0.4 μm semiporous Transwell inserts (BD Falcon^™^, San Diego, USA) in twelve-well plate. The whole procedure of establishing the model (S1 Fig) took around 30 days (Table 1) including cell expansion, co-culture the cell on the insert and culturing at air-liquid interface (S1 Information). Light-, confocal microscopy (S1 Information), scanning- and transmission electron microscopy (S1 Information) and transepithelial electrical resistance (TEER) measurement (S1 Information) were performed to characterized the morphology of differentiated PBEC under air-liquid interface (ALI) culture condition. RT-PCR was also performed to identify expression of different cell type markers (FOXJ1, MUC5AC, Club cell protein and KRT5) (S1 Information).

**Table 1:** Timeline and different procedures involved in establishing the model.

	Day 1–7	Day 8–14	Day 15	Day 16–30
PBEC expansion				
MRC-5 expansion				
Seed PBEC on inserts culture in submersion				
Add MRC-5 on other side of inserts and culture in submersion				
Culture at ALI				
Add IL-13 in airlifted medium of chronic bronchitis-like model				

In order to produce a chronic bronchitis-like mucosa, 1 ng/ml or 10 ng/ml human IL-13 (R&D SYSTEMS^®^, UK) was added to the co-culture medium when airlifted. To evaluate the effects of IL-13, RT-PCR and light microscopy were performed (S1 Information).

### Pd nanoparticles preparation and characterization

The Pd nanoparticles were prepared by solvothermal technique, exploiting the Bradley reaction, using a modified procedure [13]. Palladium acetylacetonate (99%, Aldrich CAS No 14024-61-4) *ca*. 0.2 g was dissolved in 20 ml of 99% acetophenone (>98% Aldrich CAS no 98-86-2) and subjected to reflux in 4 h. The color of the solution then changed from reddish brown to black. The formed particles were separated by centrifugation and washed by 3 portions of 10 ml 99.5% ethanol and dried in vacuum.

To characterize the Pd nanoparticles, the SEM-EDS investigation of the samples was carried out with a tabletop Hitachi TM 1000-μ-DeX scanning electron microscope. The size of individual Pd nanoparticles was established by image analysis in TEM using Philips EM 420 transmission electron microscope. The thus determined size was well correlating with that determined by Debye-Scherrer formula applied on the X-ray powder diffraction data. X-ray powder diffraction study was made with a multifunctional Bruker SMART Apex-II diffractometer operating with a MoKα radiation (l = 0.71073 Å). In solution (ethanol or KSFM medium) the hydrodynamic size of the aggregates was determined by Laser reflection microscopy (NanoSight, Malvern Instruments Ltd, Malvern, UK)

### Pd nanoparticles exposure using Xpose*ALI* (exposure system)

In order to mimic the *in vivo* exposure situation of the lung, the model was exposed to aerosolized Pd nanoparticles under ALI condition. Pd nanoparticle aerosol was generated from small batches of dry Pd nanoparticle powder using the high pressure aerosol generator of the PreciseInhale exposure platform. Triplicate model inserts were exposed to the aerosolized Pd nanoparticles at the same time using the Xpose*ALI* exposure module of the PreciseInhale system (S2A and S2B Fig). Before exposure, medium was changed and the apical side of the model was washed by PBS. Pd nanoparticles were loaded to the powder chamber of the PreciseInhale aerosol generator for each exposure cycle. Compressed air of 100–140 bars was used to aerosolize the particles into the 300 ml holding chamber. Generated aerosol was then pulled from the holding chamber through a Casella light dispersion instrument and into the exposure manifold, at a main flow rate of 90 ml/min. The main flow was diverted into three consecutive branch flows of 10 ml/min for exposure of each cell culture insert. To control the dose, the Casella signal was measured and correlated to the particles entering the exposure manifold, by calculating the substance correlation factor (scf) for the Pd nanoparticle aerosol. The scf was calculated by dividing the gravimetric deposition of Pd nanoparticle aerosol on an end filter with the integrated Casella signal from a triplicate series of test exposures only using the main flow. The scf for the Pd nanoparticles used in this study was 0.8648.

To maintain viable cells and throughout the exposures, the holding chamber was humidified by covering the inside walls with humid filter paper. During the exposures the model inserts were in contact with airlifted medium from below. Sham exposures (exposures with identical flow rate settings using only air and a clean exposure system) were done to control for potential viability effects on the model induced by the exposure itself.

3 normal and 3 chronic bronchitis-like models (model treated with 1 ng/ml IL-13; the IL-13 concentration was determined based on results obtained from RT-PCR and PAS staining) from 3 different donors were exposed for different time durations in proportion to the desired target dose (sham, low, medium and high doses). The exposure cycles took 20 s (low), 45 s (medium) and 3 min (high). Corresponding sham exposures represented all exposure times. After aerosol exposure, the models were continually incubated in 5% CO_2_ at 37°C for 8 hours or 24 hours. To get the apical medium (AM), the apical side of the epithelial layers was lavaged with 180 μl pre-heated airlifted medium for 15 minutes at room temperature. Both AM and airlifted medium from the basal chamber of the insert (BM) were collected after 8 hours or 24 hours incubation and frozen at -80°C until further use.

### Cell viability

The apical side of the insert was rinsed with 200 μl PBS to remove the mucus, then the fibroblasts were gently scraped from the model and the PBEC were trypsinized and resuspended. The viability of resuspended PBEC were detected with trypan blue to estimate the effects of both the exposure and the trypsinizing process and cell viability of more than 95% was accepted. The PBEC was then treated with annexin V—PE/7-AAD (BD Pharmingen, San Diego, USA) to detect apoptotic rate by collecting 2000 cells using flow cytometer (FACS Fortessa; BD Biosciences, San Diego, USA).

### Pd nanoparticles exposure dose and uptake

The analysis of Pd nanoparticle exposure dose in the model was carried out by an inductively coupled plasma mass spectrometer (ICP-MS) [14] using solutions produced by digestion of the model membrane with aqua regia and neutralized to pH = 3 by addition of a concentrated (25%) solution of ammonia. To identify whether the Pd nanoparticles were immobilized on the cell surface or taken up by the PBEC, the model was exposed with Pd nanoparticles for 3 min and incubated for 2, 4, 8 or 24 h post-exposure and analyzed with TEM (Supporting Information).

### Inflammatory mediators

Measurements of interleukin 8 (IL-8/CXCL8) in BM from unexposed/exposed model and AM from exposed model were performed using an in-house ELISA [15]. The detection limit of the IL-8 assay was 50 pg/ml. Levels of matrix metallopeptidase 9 (MMP-9) and club cell protein in BM from unexposed/exposed models and AM from exposed models were measured using a purchased DouSet ELISA MMP-9 Kit and uteroglobin kit (R&D SYSTEMS^®^, UK), respectively. The detection limits of the MMP-9 and club cell protein assay were 31.2 pg/ml.

### Statistics

All data were expressed as medians and interquartile ranges (25th-75th percentiles). Within model, the comparisons between different time points and different Pd nanoparticles exposure doses were assessed by Friedman test and followed by Wilcoxon signed rank t test as a *post hoc* test. The comparisons between normal, 1 ng/ml and 10 ng/ml IL-13 treated models were performed by Wilcoxon signed rank t test. A p-value <0.05 was considered as significant. All the data were analyzed using STATISTICA9 (StatSoft, Inc. Uppsala, Sweden)

## Results

### Morphological characterization of 3D model

**Fig 1:**
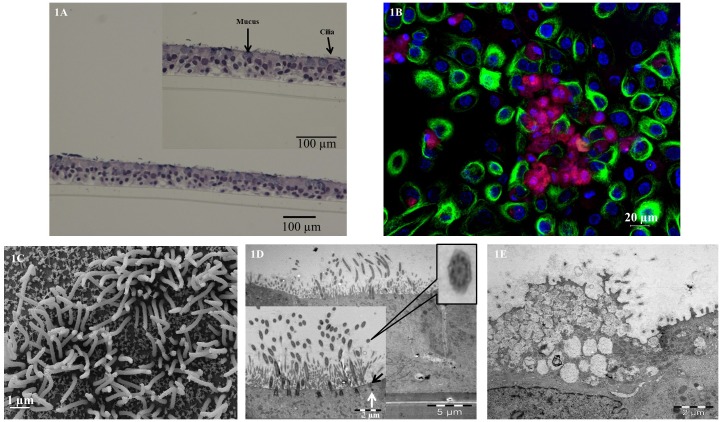
Morphological characterization of 3D model. 1A: Light microscope analysis of hematoxylin and eosin (H&E) staining of paraffin embedded cross section of 2 weeks ALI model; Bar scale: 100 μm. Higher magnification showed cilia and mucus; Bar scale: 100 μm. 1B: Confocal microscope analysis of immunofluorescence staining of ciliated cell marker anti-acetylated alpha tubulin antibody (green florescence), mucus producing cell marker anti-MUC5AC antibody (red florescence) in 2 weeks ALI model; cell nuclei stained with DAPI (blue florescence). Bar scale: 20 μm. 1C: Scanning electron microscope analysis of 2 weeks ALI model, mature cilia present; Bar scale: 1 μm. 1D: Transmission electron microscope analysis of 2 weeks ALI model, ciliated cell surface was scattered with elongated cilia; Bar scale: 5 μm. Higher magnification showed cilia displaying 9+2 axoneme formation, tight junction (black arrow) and desmosome (white arrow); Bar scale: 2 μm. 1E: Transmission electron microscope analysis of 2 weeks ALI model, mucus cell present with electron-dense cytoplasm containing electron-lucent granules; Bar scale: 2 μm.

To identify the appearance of PBEC grown under ALI culture conditions, hematoxylin and eosin (H&E) analysis of model cross sections was carried out of 3D model after culturing at ALI for 2 weeks (Fig 1A). Fig 1A showed that the PBEC were pseudo-stratified and that the model contained basal cells, mucus producing cells and ciliated cells. Due to technical problem, the fibroblast layer loosened during the embedding process, and was therefore difficult to identify. By using immunofluorescence analysis, ciliated cells and mucus-producing cells were demonstrated in the model (Fig 1B). Ciliated cells were covered by multiple cilia (green florescence), and were found together with mucus producing cells containing MUC5AC (red florescence).

To further verify the structure of the 3D models, SEM (Fig 1C) and TEM (Fig 1D and 1E) were performed. After 2 weeks cultured at ALI, the ciliated cell surface was scattered with elongated cilia. From Fig 1C, the protrusions were obvious and the maturation of cilia was prominent. In Fig 1D, the cilia of the ciliated cell showed the ultra-structure of axosome, which contains two central singlet and nine outer doublet microtubules. Basal bodies were docked to the apical cell surface. Besides the formation of tight junctions (black arrow) and desmosomes (white arrow) can be easily found. The TEER reached 192±37 Ω.cm^2^ and 187±55 Ω.cm^2^ in the normal and IL-13 treated models respectively, with no significant differences in barrier integrity between normal and IL-13 treated model (S3 Fig). From Fig 1E, the model showed a mucus producing cell where an electron-dense (high affinity) cytoplasm containing electron-lucent (low affinity) granules. At 3 and 4 weeks after being cultured at ALI, the amount of ciliated cells and the surface area carpeted by cilia began to diminish, and some of the cilia lost their normal structure and became bud-like (S4A and S4B Fig). Interestingly, we also revealed Club (Clara) cells which contain lamellar bodies (S4C Fig).

### mRNA expression of different cell type markers

**Fig 2:**
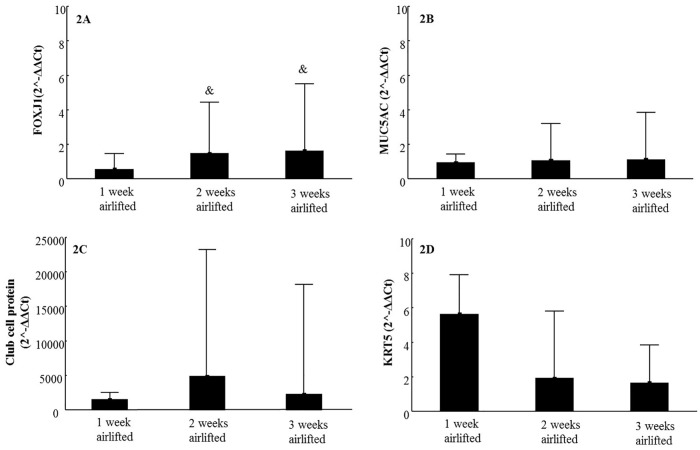
mRNA expression of different cell type markers in 3D models. Expression of ciliated cell marker FOXJ1 (2A), mucus producing cell marker MUC5AC (2B), club cell marker Club cell protein (2C) and basal cell marker KRT5 (2D) mRNA in normal models after culturing at ALI for 1, 2 and 3 weeks (N = 9). Data presented as median and 25^th^ -75^th^ percentiles; &: P<0.05 VS FOXJ1 mRNA expression in 1 week culturing at ALI.

To gain a better understanding of PBEC differentiation after ALI culturing, we investigated the mRNA expression of different cell type specific markers: FOXJ 1 (ciliated cell), MUC5AC (mucus producing cell), Club cell protein (club cell) and KRT5 (basal cell), after being cultured for 1, 2 and 3 weeks at ALI (Fig 2A–2D). According to Fig 2A, the mRNA expression of FOXJ1 was significantly higher after being airlifted for 2 or 3 weeks compared to 1 week. The mRNA expression of MUC5AC (Fig 2B), Club cell protein (Fig 2C) and KRT5 (Fig 2D) did not change significantly over time.

### Effects of IL-13 stimulation

**Fig 3:**
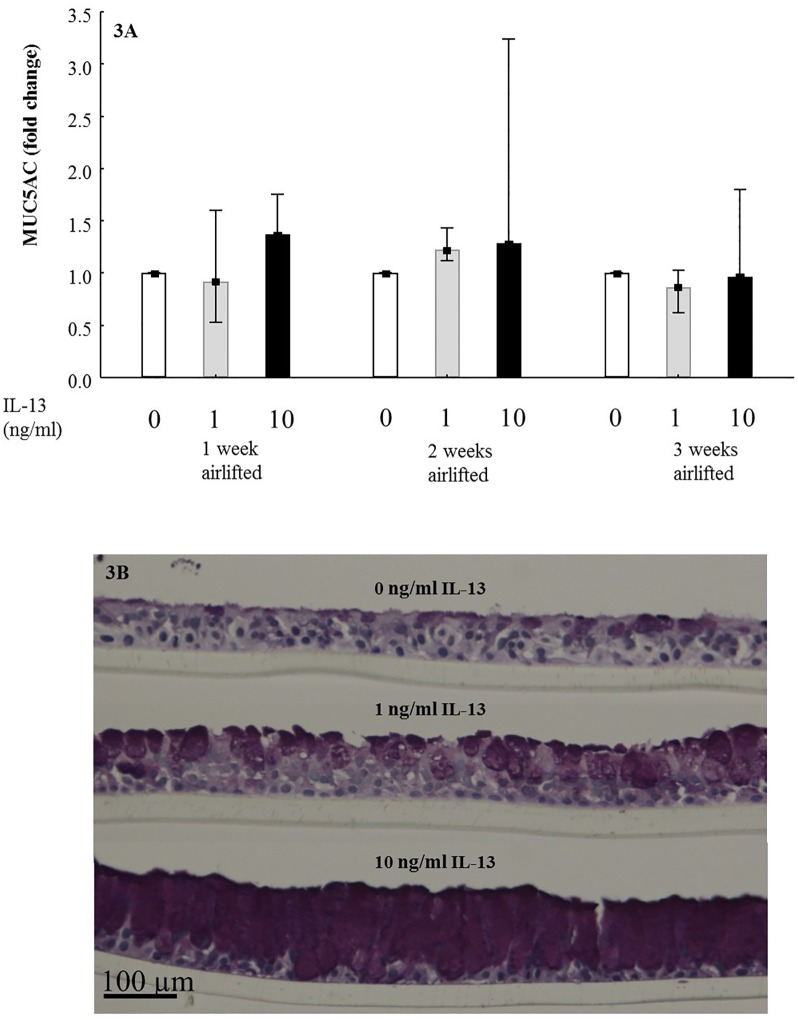
Effects of IL-13 stimulation on 3D models. 3A: Fold change of MUC5AC mRNA expression in ALI models treated without (blank) and with 1 ng/ml (grey) and 10 ng/ml (black) IL-13 for 1, 2 and 3 weeks (N = 9); Data presented as median and 25^th^ -75^th^ percentiles. 3B: Periodic acid—Schiff (PAS) staining of paraffin embedded cross section of ALI models treated without and with 1 ng/ml and 10 ng/ml IL-13 for 2 weeks visualized by light microscope; Bar scale: 100 μm.

The model was treated with different concentrations of IL-13 at different time points. Fig 3A, S5A and S5B Fig showed the fold changes of MUC5AC, FOXJ1 and Club cell protein mRNA expression after treatment of 1 or 10 ng/ml IL-13 compare with untreated models. After 1 and 2 weeks of culturing at ALI, IL-13 tended to increase MUC5AC expression compared to untreated model although not significantly (Fig 3A). As for FOXJ1, there is no clear effect at different time points or different concentrations of IL-13 treatment (S5A Fig). At all three time points, the mRNA expression of Club cell protein showed a significantly IL-13 concentration dependent decrease (S5B Fig).

From Fig 3B, using light microscope we identified a dose dependent effect of IL-13 treatment, more mucus producing cells present in the model from higher concentrations of IL-13 after 2 weeks culturing at ALI.

### Pd nanoparticles characterization

**Fig 4:**
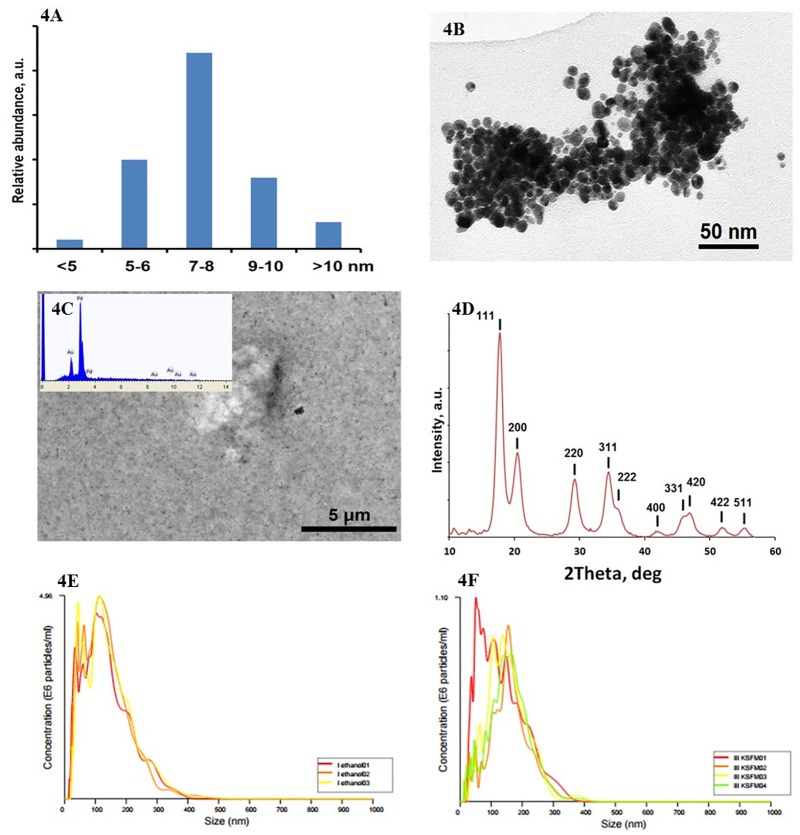
Characterization of Pd nanoparticles. 4A: Distribution of Pd nanoparticles size; 6–10 nm with a distinctly pronounced maximum at 8 nm. 4B: Transmission electron microscope views of the produced Pd nanoparticles; Bar scale: 50 nm. 4C: Scanning electron microscope view of the Pd nanoparticles collected on a gold-sputtered carbon filter with an EDS spectrum as in set; Bar scale: 5 μm. 4D: X-ray powder diffraction pattern of the produced Pd nanoparticles. 4E: Hydrodynamic size of the aggregates in ethanol; determined by Laser reflection microscopy; 138±3 nm. 4F: Hydrodynamic size of the aggregates in KSFM medium; determined by Laser reflection microscopy; 151±6 nm.

The synthesized Pd nanoparticles were uniform spherical objects with a narrow size distribution (6–10 nm with a distinctly pronounced maximum at 8 nm) (Fig 4A). The particles were agglomerated when drying (Fig 4B), but easily dispersed by sonication and formed optically uniform sols or aerosols (in air). When collected on a gold-covered carbon filters they appeared as an extremely fine powder with single particles non-distinguishable by SEM. The EDS analysis demonstrated a high purity of the obtained Pd (Fig 4C). The X-ray diffraction proved that the obtained material was reduced Pd metal as the observed lines were all in good agreement with reference values (JCPDS card No. 87–0638) (Fig 4D). The hydrodynamic size of the aggregates in ethanol (Fig 4E) and KSFM medium (Fig 4F) were 138±3 and 151±6 nm, respectively.

### Pd nanoparticles exposure and uptake

**Fig 5:**
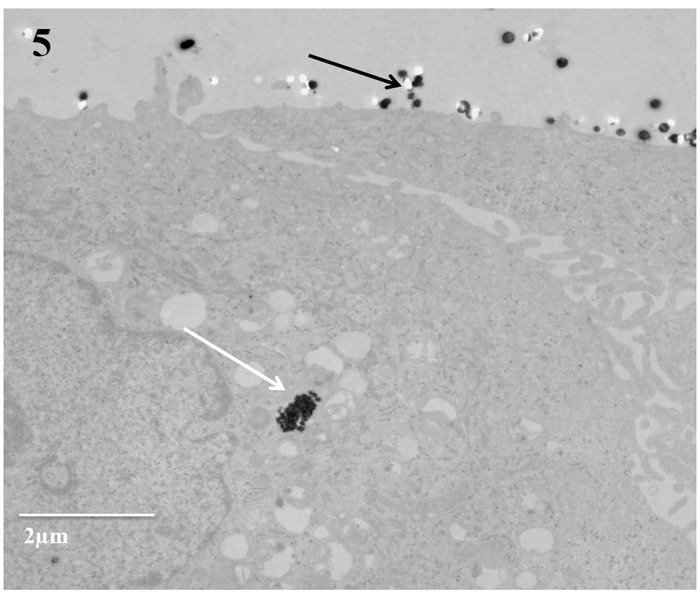
Uptake of Pd nanoparticles. The internalization of Pd nanoparticles in 3 mins exposed 3D model and incubated for 24 hours post exposure; most of Pd nanoparticles localized on the cell surface (black arrow), while few Pd nanoparticles localized inside the cells (white arrow); Bar scale: 2 μm.

The models were exposed to three doses of Pd nanoparticles: 250 ng/cm^2^ (low), 400 ng/cm^2^ (medium) and 650 ng/cm^2^ (high). We observed that most of Pd nanoparticles immobilized on the cell surface while there were just a few Pd nanoparticles found within the cells incubated for 24 hours after exposure and there were no differences between different time points (Fig 5). According to S6 Fig, after the initial deposition (S6A Fig), Pd nanoparticles were found to be redistributed and accumulated at one spot (S6B Fig).

### Cell viability

The viability of the model was more than 95% both before and after exposure. The apoptotic rate was 1% to 30% and there are no differences between sham and Pd nanoparticles exposure of either the normal and IL-13 treated models.

### Inflammatory mediators in unexposed models

The levels of soluble IL-8, Club cell protein and MMP-9 in the BM of unexposed models after 24 hours incubation are shown in Table 2. The release of IL-8 in IL-13 treated model was significantly higher than in the normal model, and the release of Club cell protein showed the same trend, although not significant. The MMP-9 levels in both models were in the same range in both types of models.

**Table 2:** Inflammatory mediators from basal medium after 24 hours incubation of unsponsored model.

	Normal model	Chronic bronchitis-like model
**IL-8 (ng/ml)**	22.28 (7.04–38.14)	39.13**(9.42–69.53)
**Club cell protein (ng/ml)**	3.72 (2.65–15.63)	18.80 (6.51–22.37)
**MMP-9 (ng/ml)**	15.71 (14.44–18.74)	11.95 (4.98–13.08)

3 normal and 3 chronic bronchitis-like models from 3 different donors, N = 9

Normal model: non-IL-13 treated model. Chronic bronchitis-like model: 1 ng/ml IL-13 treated model.

Results are presented as median and 25^th^ -75^th^ percentiles.

** indicate P<0.01, comparisons between normal and chronic bronchitis-like model (Wilcoxon signed rank t test).

### Inflammatory mediators in Pd nanoparticles exposed models

**Fig 6:**
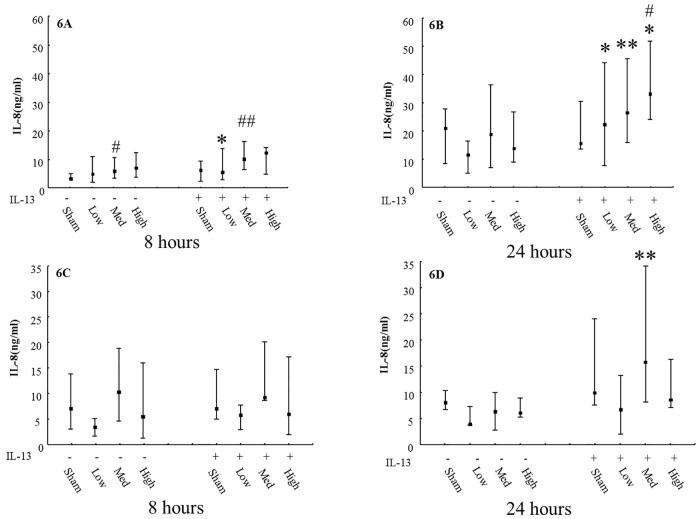
Release of IL-8 after Pd nanoparticles exposures using the Xpose*ALI* system. Levels of IL-8 in basal medium in normal or 1 ng/ml IL-13 treated models (N = 9) after exposure to different exposure doses of Pd nanoparticles and incubated for 8 hours (6A) and 24 hours (6B). Levels of IL-8 in apical medium in normal or 1 ng/ml IL-13 treated models (N = 9) after exposure to different exposure doses of Pd nanoparticles and incubated for 8 hours (6C) and 24 hours (6D). Exposure: sham: normal air and clean system; low: 250 ng/cm^2^; med: 400 ng/cm^2^; high: 650 ng/cm^2^. Data presented as median and 25^th^ -75^th^ percentiles; #: P<0.05 VS Sham exposure; *, **: P<0.05, 0.01 VS normal model.

Concentrations of IL-8 levels in BM and AM from the models after exposure are shown in Fig 6. At 24 hours of incubation after Pd nanoparticle exposure, the chronic bronchitis-like model secreted more IL-8 than the normal model (P<0.05). In BM, the IL-8 level was higher after 24 hours incubation than after 8 hours, while in AM, there was no difference between these two time points (Fig 6C and 6D).

Regarding release of MMP-9 and Club cell protein, there was no significant difference between normal and chronic bronchitis-like model and no significant difference between different Pd nanoparticles exposure doses., For all models, the level of MMP-9 secretion in AM was significantly higher than in BM (P<0.05) (S7 Fig).

## Discussion

In this study, we succeeded in combining 3D models mimicking both healthy and chronic bronchitis-like mucosa (by using PBEC and fibroblast) which consisted of all types of cells present*in vivo* with controlled aerosol exposure module (Xpose*ALI*). In order to evaluate this combined system, exposure to Pd nanoparticles induced an increased secretion of IL-8, where chronic bronchitis-like model released significantly more IL-8 than normal models.

*In vivo*, human bronchial epithelium consists of 50–70% ciliated cells, up to 30% basal cells, up to 25% goblet cells, and 11% Club (Clara) cells [16]. When culturing at ALI the epithelial cells differentiated and we have identified all cell types listed above by light/confocal microscope, SEM, TEM, mRNA expression, and protein secretion. The FOXJ1expression increased in our models after 2 and 3 weeks of culturing at air-liquid interface compared with week 1, indicating that the number of ciliated cell increased over time.

According to previous studies, *in vitro* stimulation with IL-13 induces mucus producing cell hyperplasia which is a characteristic feature of chronic bronchitis [17]. The effects of different concentrations of IL-13 in human cell culture model varied [10, 18]. Based on PAS stained cross sections, we identified that at 2 weeks culturing at ALI, the model treated with 1 ng/ml IL-13 have more mucus producing cells than untreated model. However, after 2 weeks culturing at ALI, in the models treated with 10 ng/ml IL-13 almost all cells were mucus producing cells as identified by PAS stained cross sections, while the models treated with 1 ng/ml IL-13 showed different cell types, although more mucus producing cells compared with untreated models. Moreover, MUC5AC mRNA expression was slightly increased after IL-13 treatment for 2 weeks at ALI conditions. These findings indicate that the model which mimics chronic bronchitis-like mucosa was created after stimulation with 1ng/ml IL-13 and cultured at ALI for 2 weeks.

In the present study, we succeeded in combining the 3D models with controlled aerosol exposures using the Xpose*ALI* exposure module and thereby managed to deposit nanoparticles on a well differentiated cellular layer cultured at ALI. There are some commercially available cell exposure systems, such as ALICE, Vitrocell and Cultex^®^ RFS [19–21]. It was an important breakthrough when *in vitro* inhalation research was able to expose ALI cells to aerosols in a manner resembling *in vivo* exposures with aerosols and particles deposited directly onto lung cells. In this study, the unique performance of the Xpose*ALI* exposure module was linked to its close integration with the PreciseInhale aerosol generator and dosing system [11, 12]. The aerosolization in the PreciseInhale system was driven by compressed air with pressures up to 160 bar generating well dispersed particles through deagglomeration of loaded powder portions. Also, nanoparticle powders with high cohesive energies were generally well dispersed by the PreciseInhale system. Therefore, the amounts of powdered substance required were minimal. A key feature of the combined exposure system was the dosing technics including the Casella light dispersion that automatically controls the dose drawn from the generator to the cell surface and steered by a computer at each experiment. Another advantage of the combined exposure system was that Xpose*ALI* generated more concentrated aerosols which gave shorter exposure times. Therefore, it minimized the time for cells being out of the incubator, which is an important survival factor for human primary cells. In addition, sufficient deposition of aerosol in the 3D models can be achieved even without using more complex methods such as electrostatic deposition. In the combined 3D cell models cultured in inserts and Xpose*ALI* exposure system, the exposure hoods restrict the area available for particle exposure to only the PBEC surface (not the insert walls) and the particles can be evenly distributed over the whole PBEC surface. By using a reverse flow pattern over the PBEC surface from the periphery to one central outlet, excess aerosol can be collected in filters located on top of the exposure hoods, which reduce the risk of an unhealthy laboratory environment.

For both healthy subjects, and subjects with respiratory diseases, our chosen test substrates of highly dispersed Pd nanoparticles present in polluted ambient air are of clinical importance. Pd nanoparticle is a traffic-related particulate pollution, originating from the emission of particles constituting the active component of the catalytic converters in road vehicles [22]. The complex particles leaving the catalytic converters are about 10 nm in size resulting in a relative stable aerosol that usually deposit in the deep lung such as the alveolar region, but the deposition will also occur higher up in the airway tree particularly at the bifurcations [23]. The synthesized Pd nanoparticles in this study were uniform about 6–10nm in size, which could represent *in vivo* situation.

After the exposure, levels of IL-8 from both AM and BM were detected. IL-8 is an important chemoattractant and activator which is produced by airway epithelial cells. Increased level of IL-8 has been identified in different compartments in smokers with and without COPD [24]. Moreover, IL-8 secretion has been well studied as an inflammatory biomarker in response to particle exposures [25]. In this paper, we found in the BM of Pd nanoparticles exposed models, the levels of IL-8 were significantly higher in chronic bronchitis-like model than in normal model after 24 hours of incubation and showed the same tendency after 8 hours of incubation. Also in unexposed models, IL-13 treated models secreted significantly more IL-8 than non-13 treated model. These results may indicate that the chronic bronchitis-like model is more sensitive to Pd nanoparticles exposure than the normal model. Besides, individuals with COPD have been shown to have an increased sensitivity to particulate matter in air pollution [26] which confirms that our combined exposure system *in vitro* can emulate the real situation *in vivo*.

In our previous study, we demonstrated that submerged PBEC cultures exposed to medium dispersed Pd nanoparticles secretion of IL-8 tend to increase at higher Pd nanoparticle concentration [13]. In line with this finding, we showed that Pd nanoparticle exposures dose-dependently increased the release of IL-8 in BM of both the normal and chronic bronchitis-like models. These results may demonstrate that exposure to Pd nanoparticles can cause inflammatory response in both normal and chronic bronchitis-like models after 8 hours, and the effects of Pd nanoparticles exposure were increased with the dose. After 24 hours incubation, the influence of Pd nanoparticles exposure on IL-8 level disappeared in the normal model, but still existed in the chronic bronchitis-like model. This may occur because of the normal model being able to self-regulate and recover from inflammation caused by Pd nanoparticles exposures after 24 hours. However, for the chronic bronchitis-like model, the recovery process may not be as efficient as in the normal model and may need longer time.

MMP-9 is a type IV collagenase that is related to remodeling [24] and will not be produced by resident lung cells until lesions occur to the bronchial surface in form of mechanical trauma or exposure to toxic substances or inflammatory mediators. Metaplastic epithelial cells produce the highest amounts of MMP-9, whereas production by fibroblasts is minimal [26]. We found that levels of MMP-9 in AM were much higher than in BM. This difference was observed despite the fact that the AM was obtained by a 15 min lavage periods of the 3D models’ apical surface at 8 or 24 hours after exposure, while the samples of BM contained the cumulative secretion of MMP-9 for 8 or 24 hours. These results suggest that Pd nanoparticles caused epithelial MMP-9 secretion as response to damage that requires extracellular matrix (ECM) remodeling, probably for quick re-epithelialization [27]. These results were also in contrast to IL-8 levels, as there were no differences in IL-8 levels between BM and AM. This might be due to size differences as the molecular weight of IL-8 (8.4 kDa) is much smaller than of MMP-9 (92 kDa). Therefore, IL-8 could more easily penetrate through confluent cell layer and the insert membrane. Besides, from the basal fibroblasts the contribution to the basal medium were probably higher from IL-8 compared to MMP-9 [28]. A third explanation for this could also be that there were differences in the direction of secretion between IL-8 and MMP-9.

Analysis of the models with TEM showed very little uptake of the particles by the PBEC. This was in marked contrast to our previous study using the same cells and particles, but with cells cultured under submerged condition and particles dispersed in cell culture medium [13]. When the particles were added to the medium, an uptake was observed already within 1 hour [13]. The lack of uptake in our 3D model cultured under ALI conditions could be explained by the presence of mucociliary clearance. After the initial deposition, particles were found to be redistributed and accumulated at one spot which probably was caused by the beating of cilia. The lack of a protective mucus layer in the submerged culture could also explain the difference in uptake of Pd nanoparticles by PBEC.

## Conclusion

In present study, we succeeded in combining relevant human airway wall models with controlled aerosol exposures using the Xpose*ALI* exposure module. In addition, the modification of the 3D model leading to mucus producing cell metaplasia and hyperplasia, which are phenomena observed in airway diseases such as COPD and chronic bronchitis, are suited for mimicking interactions between nanoparticle exposures and the innate immune response in chronic bronchitis patients. For both healthy subjects, and subjects with respiratory diseases, our chosen test substrate, Pd nanoparticles present in polluted ambient air, is of clinical importance. With further development and validation, this exposure setting could form part of an *in vitro* testing strategy to reduce the requirement for animal inhalation studies.

## Supporting Information

S1 Fig A diagrammatic representation of the main steps in establishing the model. S1A: Apical seeding of PBEC into a 0.4 μm semiporous transwell insert; S1B: Basolateral seeding of fibroblast; S1C: Removed medium and only added airlifted medium in the basolateral chamber; S1D: Differentiation when culturing at ALI. All steps were performed under sterile conditions and cells were cultured at 37°C and 5% CO_2_.
(TIF)

S2 Fig The Xpose*ALI* exposure system. The Xpose*ALI* cell exposure module (S2A) and a close up of the transwell insert with cells (down), the exposure hood (middle) and the exposure filter holder (up) (S2B).
(TIF)

S3 Fig Transepithelial electrical resistance (TEER) of 3D model. Transepithelial electrical resistance (TEER) of normal (blank) and 1ng/ml IL-13 treated (black) models. Data present as median and 25th -75th percentiles.
(TIF)

S4 Fig Morphological characterization of 3D model. S4A &S4B: Scanning electron microscope analysis of 3 and 4 weeks ALI model, cilia begin to lost their normal structure and became bud-like; Bar scale: 1 μm. S4C: Transmission electron microscope analysis of 2 weeks ALI model, club cell which contain lamellar bodies was present; Bar scale: 2 μm. Higher magnification showed concentric/parallel lamellae, Bar scale: 1 μm.
(TIF)

S5 Fig Effects of IL-13 stimulation on 3D models. S5A & S5B: Fold change of FOXJ1 and club cell protein mRNA expression in ALI models treated without (blank) and with 1 ng/ml (grey) and 10 ng/ml (black) IL-13 for 1, 2 and 3 weeks (N = 9); Date present as median and 25^th^ -75^th^ percentiles. ‡,‡‡: P<0.05, 0.01 VS Club cell protein mRNA expression in non-IL-13 treated models. tm¤: P<0.01 VS Club cell protein mRNA expression in 1ng/ml IL-13 treated models.
(TIF)

S6 Fig Illustration of mucociliary clearance. S6A: The models were exposed to high dose (650 ng/cm^2^) of Pd nanoparticles and even distribution was observed. S6B: The Pd nanoparticle redistributed and accumulated at one spot within 24 hours, indicating existing mucociliar clearance.
(TIF)

S7 Fig Release of MMP-9 after Pd nanoparticles exposures using the Xpose*ALI* system. Levels of MMP-9 in basal medium in normal or 1 ng/ml IL-13 treated models (N = 9) after exposure to different deposited doses of Pd nanoparticles and incubated for 8 hours (S7A) and 24 hours (S7B). Levels of MMP-9 in apical medium in normal or 1 ng/ml IL-13 treated models (N = 9) after exposure to different concentrations of Pd nanoparticles and incubated for 8 hours (S7C) and 24 hours (S7D). Exposure: sham: normal air and clean system; low: 250 ng/cm^2^; med: 400 ng/cm^2^; high: 650 ng/cm^2^. Data presented as median and 25^th^ -75^th^ percentiles; ##: P<0.01 VS Sham exposure; *: P<0.05 VS normal model.
(TIF)

S1 Information Supporting text. (DOCX)
