# Supplementary figures and images for: Development of Combining of Human Bronchial Mucosa Models with XposeALI® for Exposure of Air Pollution Nanoparticles
Source: PLoS One. 2017 Jan 20;12(1):e0170428. doi: 10.1371/journal.pone.0170428 (PMC5249057; doi:10.1371/journal.pone.0170428)

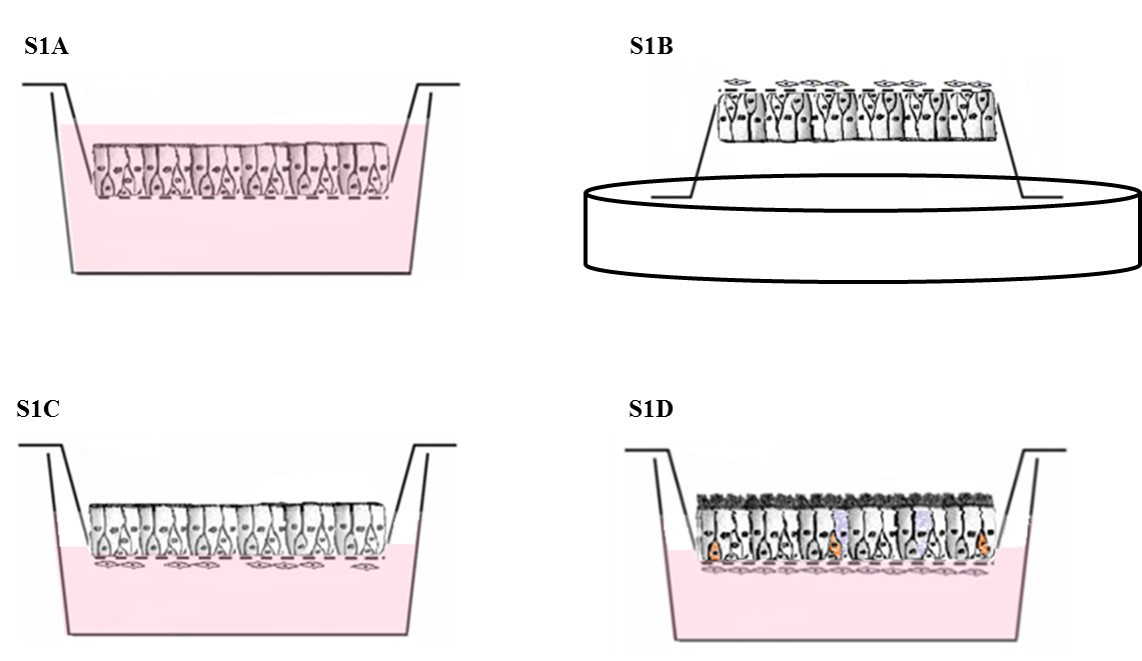

Supplement: S1 Fig — S1A: Apical seeding of PBEC into a 0.4 μm semiporous transwell insert; S1B: Basolateral seeding of fibroblast; S1C: Removed medium and only added airlifted medium in the basolateral chamber; S1D: Differentiation when culturing at ALI. All steps were performed under sterile conditions and cells were cultured at 37°C and 5% CO2. (TIF) [file pone.0170428.s001.tif]

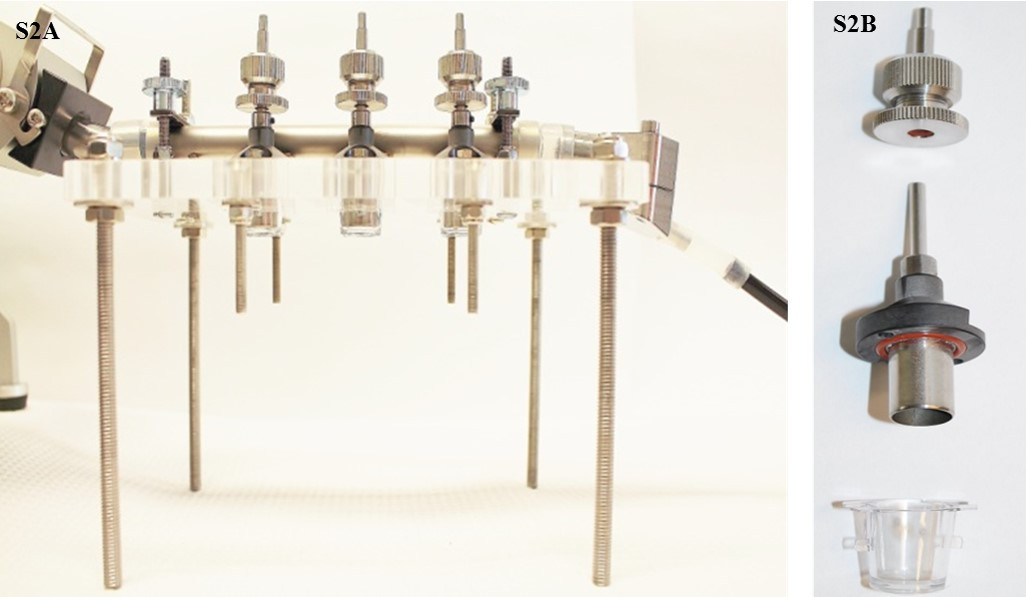

Supplement: S2 Fig — The XposeALI cell exposure module (S2A) and a close up of the transwell insert with cells (down), the exposure hood (middle) and the exposure filter holder (up) (S2B). (TIF) [file pone.0170428.s002.tif]

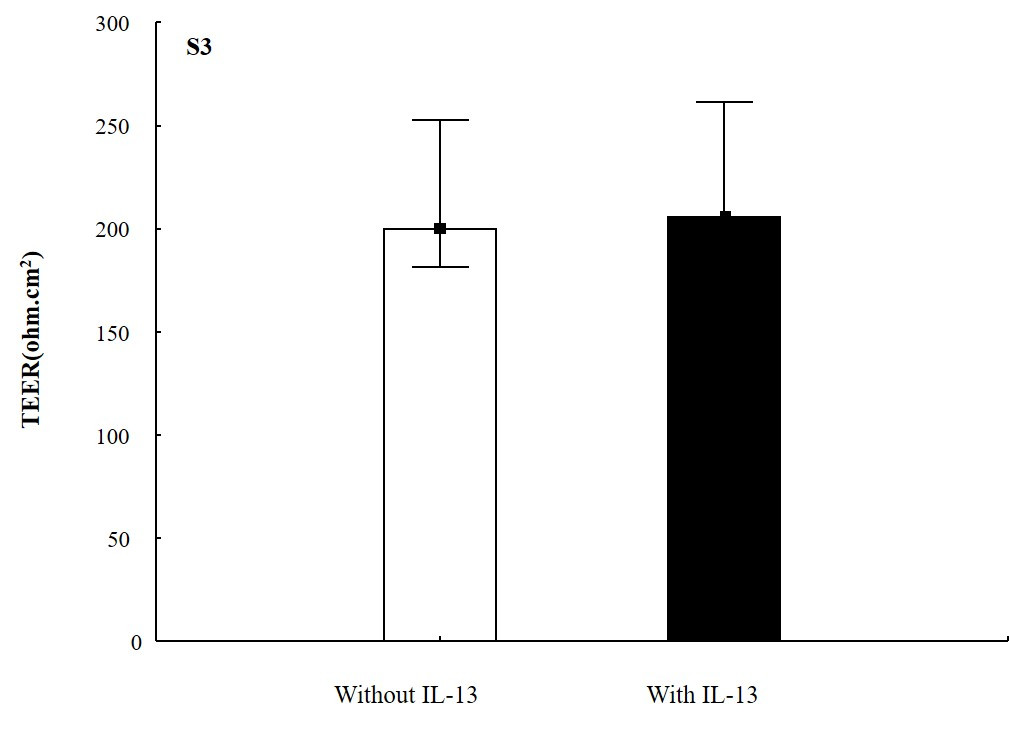

Supplement: S3 Fig — Transepithelial electrical resistance (TEER) of normal (blank) and 1ng/ml IL-13 treated (black) models. Data present as median and 25th -75th percentiles. (TIF) [file pone.0170428.s003.tif]

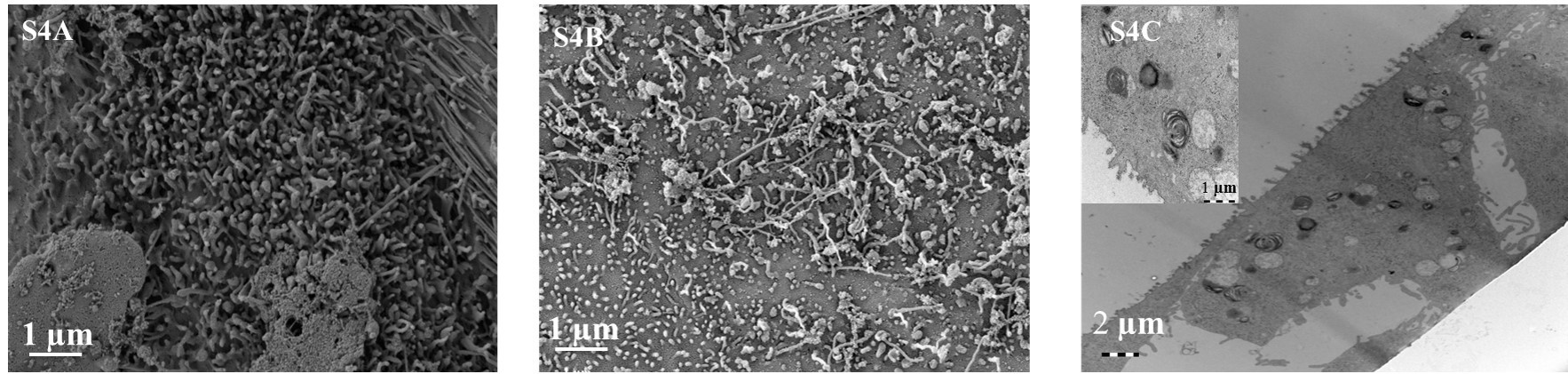

Supplement: S4 Fig — S4A &S4B: Scanning electron microscope analysis of 3 and 4 weeks ALI model, cilia begin to lost their normal structure and became bud-like; Bar scale: 1 μm. S4C: Transmission electron microscope analysis of 2 weeks ALI model, club cell which contain lamellar bodies was present; Bar scale: 2 μm. Higher magnification showed concentric/parallel lamellae, Bar scale: 1 μm. (TIF) [file pone.0170428.s004.tif]

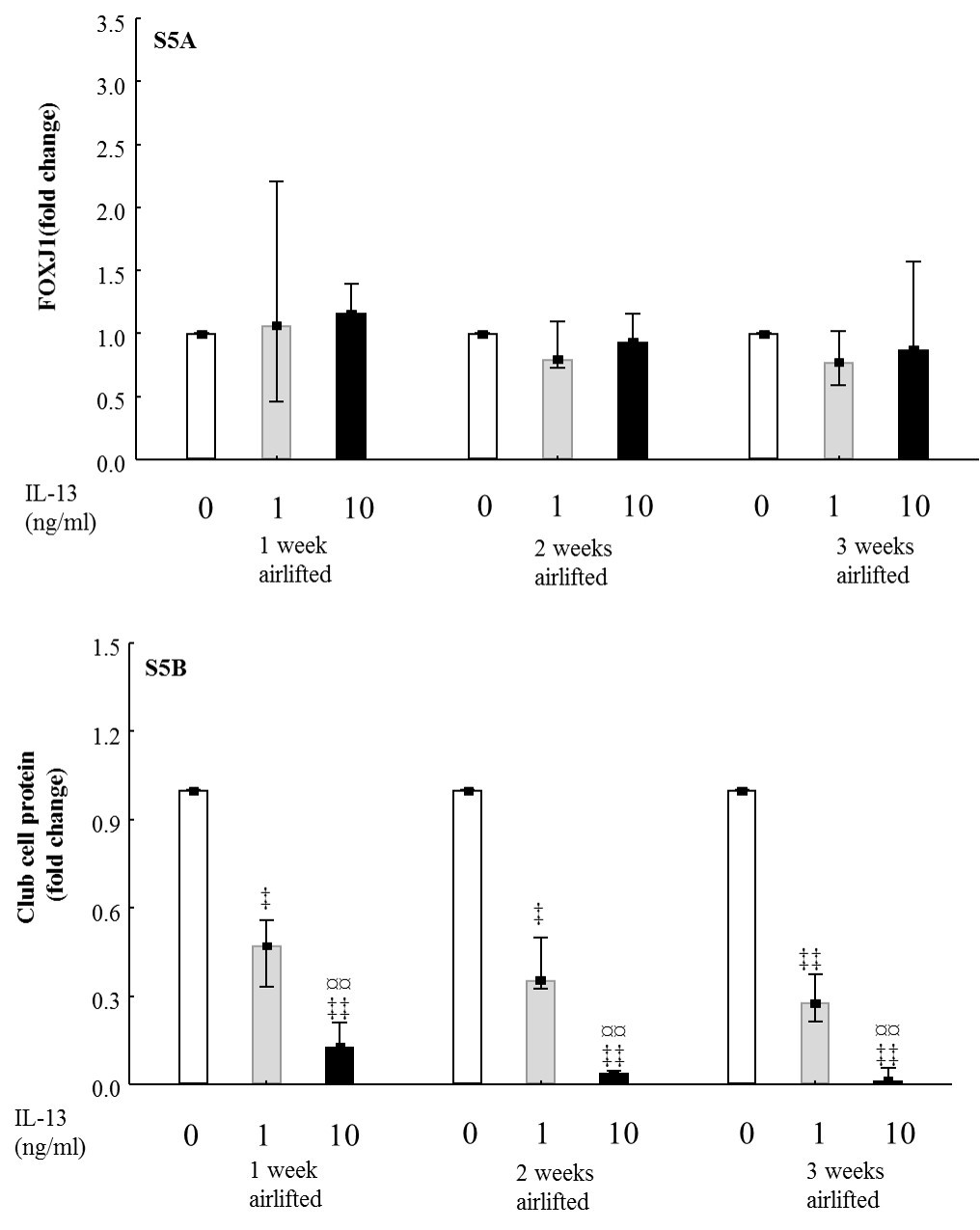

Supplement: S5 Fig — S5A & S5B: Fold change of FOXJ1 and club cell protein mRNA expression in ALI models treated without (blank) and with 1 ng/ml (grey) and 10 ng/ml (black) IL-13 for 1, 2 and 3 weeks (N = 9); Date present as median and 25th -75th percentiles. ‡,‡‡: P<0.05, 0.01 VS Club cell protein mRNA expression in non-IL-13 treated models. tm¤: P<0.01 VS Club cell protein mRNA expression in 1ng/ml IL-13 treated models. (TIF) [file pone.0170428.s005.tif]

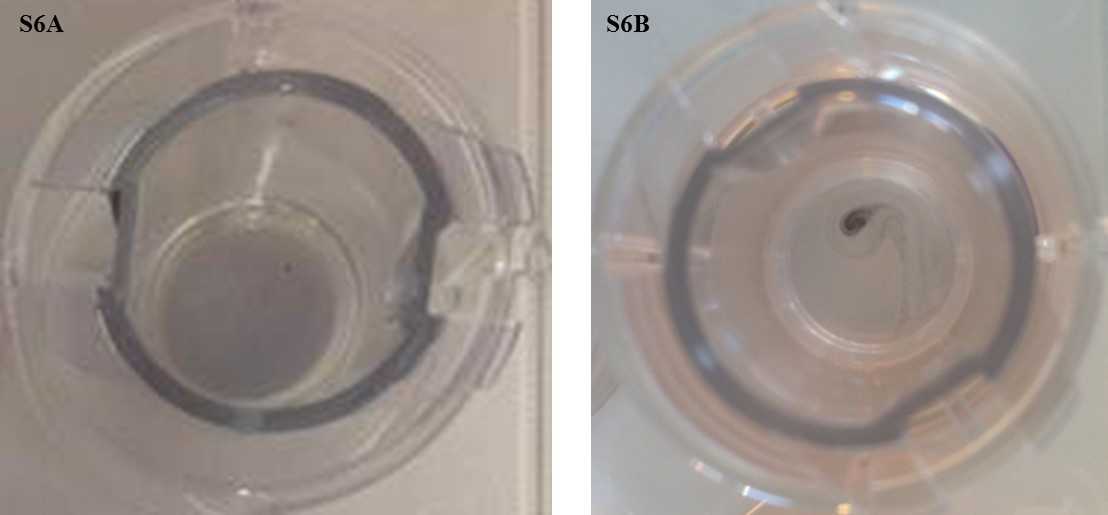

Supplement: S6 Fig — S6A: The models were exposed to high dose (650 ng/cm2) of Pd nanoparticles and even distribution was observed. S6B: The Pd nanoparticle redistributed and accumulated at one spot within 24 hours, indicating existing mucociliar clearance. (TIF) [file pone.0170428.s006.tif]

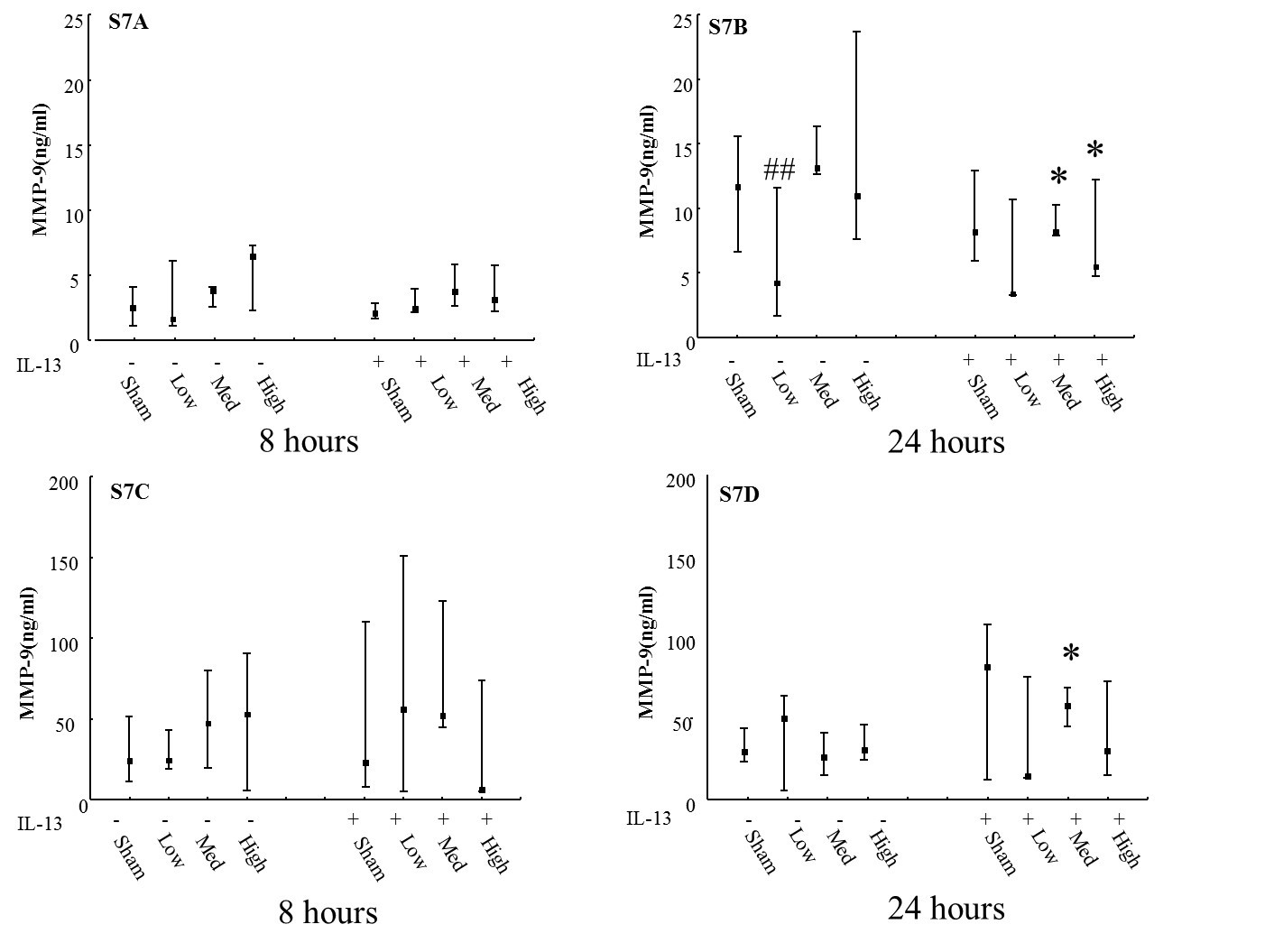

Supplement: S7 Fig — Levels of MMP-9 in basal medium in normal or 1 ng/ml IL-13 treated models (N = 9) after exposure to different deposited doses of Pd nanoparticles and incubated for 8 hours (S7A) and 24 hours (S7B). Levels of MMP-9 in apical medium in normal or 1 ng/ml IL-13 treated models (N = 9) after exposure to different concentrations of Pd nanoparticles and incubated for 8 hours (S7C) and 24 hours (S7D). Exposure: sham: normal air and clean system; low: 250 ng/cm2; med: 400 ng/cm2; high: 650 ng/cm2. Data presented as median and 25th -75th percentiles; ##: P<0.01 VS Sham exposure; *: P<0.05 VS normal model. (TIF) [file pone.0170428.s007.tif]
