# Supplementary material for: Development of Combining of Human Bronchial Mucosa Models with XposeALI® for Exposure of Air Pollution Nanoparticles
Source: PLoS One. 2017 Jan 20;12(1):e0170428. doi: 10.1371/journal.pone.0170428 (PMC5249057; doi:10.1371/journal.pone.0170428)
Supplement: S1 Information — (DOCX) [file pone.0170428.s008.docx]

**S1 Supporting Information**

**Cells**

The PBEC were harvested from healthy bronchial tissues obtained from 3 donors in connection with lobectomy. All donors gave their informed and written consent and the study was approved by the Ethics Committee of Karolinska Institutet, Stockholm, Sweden. According to the ethics statement, no specific information can be collected from the donors. Such cells have been used in different studies [1, 2] and are well characterized. The PBEC were cultured on petri dishes (ThermoFisher Scientific, Massachusetts, USA) precoated with coating buffer (fibronectin (1 mg/ml, Gibco, Life technologies, Paisley, UK), bovine serum albumin fraction V (BSA; 1 mg/ml; Sigma, Germany), vitrogen 100 collagen (3.1 mg/ml; Cohesion Technologies, Palo Alto, USA) and PBS without Ca^2+^/Mg^2+^ (Life technologies, Paisley, UK)) and maintained in Keratinocyte serum-free medium (KSFM; Life technologies, Paisley, UK.) supplied with human recombinant epidermal growth factor (EGF; 5 ng/ml; Life technologies, Paisley, UK), bovine pituitary extract (BPE; 50 µg/ml; Life technologies, Paisley, UK), penicillin streptomycin antibiotics (PEST, 1 %; Lonza, Basel, Switzerland) and retinoic acid (RA; 10^-5^M; Sigma, Germany). The cells were used at passage 3.

MRC-5 is a human fetal lung fibroblasts cell line (American Type Culture Collection ATCC, Manassas, VA), frozen at passage 24. They were seeded on tissue-culture flask (ThermoFisher Scientific) and maintained in Dulbecco's modified eagle medium (DMEM; Life technologies, Paisley, UK) with HEPES (1 %; Life technologies, Paisley, UK), nonessential amino acids (1 %; Sigma, Germany), penicillin streptomycin antibiotics (1 %; Lonza, Basel, Switzerland) and heat-inactivated FBS (10 %; Life technologies, Paisley, UK). The cells were used at passage 26.

**3D model preparation**

The main steps in establishing our models is illustrated in S1 FigTo set up the model, PBEC were seeded at 1×10^5^ cells/cm^2^ and cultured on apical side of the transwell insert precoated for 2 hours with coating buffer. Then 1 ml complete KSFM medium (with all supplements) were added to both basal and apical side of the insert. In the following week, culture medium was replaced every second day. After 1 week, the number of cells reached around 3×10^5^ cells/cm^2^, and all culture medium was removed, the insert was turned upside down and placed in a sterile petri dish. Then 100 µl of complete DMEM medium (with all supplements) containing 1×10^4^ cells/ml of fibroblasts, serving both as stromal cells and a feeding layer were added to the downside of the insert membrane. The petri dish was covered and incubated for 30 min at 37 °C; 50 µl complete DMEM was added every 10 min to prevent draining. After a total of 1 h incubation, the insert was put back into the twelve-well plates in normal position with 1 ml complete KSFM medium inside and outside the insert. The model was cultured submerged overnight in 5% CO_2_ at 37 ºC to allow the two cell types adapt to each other. To culture the models at air-liquid-interface (ALI), all the medium was removed and 800 µl airlifted medium were added (complete KSFM medium supplied with CaCl_2_ in ddH2O (6 µg/ml; Sigma, Germany), ethanolamine in ddH_2_O (15 ng/ml; Sigma, Germany) and retinoic acid (RA; 10-^5^ M; Sigma, Germany)) only in the basolateral chamber to let the apical chamber be exposed to the air. The model can be kept in 5 % CO_2_ at 37 ºC up to 4 weeks by changing the airlifted medium in the basolateral chamber every second day. After 3 weeks of culturing at ALI, the numbers of cells in our models were able to reach around 1.4-1.8×10^6^ cells/cm^2^.

To determine the effects of IL-13, the models were stimulated by addition of 1 ng/ml and 10 ng/ml recombinant human IL-13 (R&D SYSTEMS^®^, UK) to the airlifted medium. All the other procedures were the same as above.

**Histological and immunofluorescence analysis**

For histological analysis, model membranes were cut from insert and fixed in 4 % formalin overnight. After dehydration in graded ethanol, the membranes were embedded in paraffin and sectioned at 5 µm thickness. The sections were stained with Hematoxylin and Eosin (H&E) or Periodic acid–Schiff (PAS). The staining profiles in the sections were captured and visualized using BX50 light microscope (Olympus Optical Co., Tokyo, Japan).

For immunofluorescence analysis, the 4 % formalin fixed model membranes were first washed by PBS and blocked by PBS with 1 % goat serum (R&D SYSTEMS^®^, UK). Then the membranes were stained with primary antibodies: mouse anti-acetylated alpha tubulin antibody (1:200; abcam, Cambridge, UK) and rabbit anti-mucin 5AC antibody (1:100; abcam, Cambridge, UK) followed by secondary antibodies: Alexa Fluor ^®^ 488-conjugated goat anti-mouse IgG (1:800; abcam, Cambridge, UK), Alexa Fluor ^®^ 555-conjugated goat anti-rabbit IgG (1:400; abcam, Cambridge, UK) and mounted on microscope slides with DAPI (abcam, Cambridge, UK). Negative control slide was also prepared by excluding the primary antibodies. Images were captured and visualized using a LSM700 confocal microscope (Zeiss, Germany).

**Scanning and transmission electron microscopy**

The membranes of the model were cut from the insert and fixed in 2% glutaraldehyde in 0.1 M sodium cacodylate buffer containing 0.1 M sucrose and 3 mM CaCl_2_ (pH=7.4). The fixed membranes were dehydrated in graded ethanol, and dehydration were completed by critical point drying under CO_2_. Then the membranes were mounted and sputter-coated for examining in a scanning electron microscope.

The membranes of the model were cut from the insert and fixed in 2% glutaraldehyde in 0.1 M sodium cacodylate buffer containing 0.1 M sucrose and 3 mM CaCl_2_ (pH=7.4). Postfixation of the membranes were performed in 2 % osmium tetroxide in 0.07 M sodium cacodylate buffer (pH=7.4) for 2 hours. Then the membranes were dehydrated in ethanol followed by acetone, and embedded in LX-112 (Ladd, Burlington, USA). After sectioning on an ultramicrotome, the sections were contrasted with uranyl acetate followed by lead citrate and examined in a Tecnai 12 transmission electron microscope.

**Transepithelial electrical resistance (TEER) measurement**

The transepithelial electrical resistance (TEER) was measured with an EVOM voltage-ohm meter and chopstick electrodes (World Precision Instruments, New Haven, USA). The TEER values were calculated from the mean of 8 inserts per condition, subtracting the TEER value from an insert without cells and multiplying the result by the effective growth area of the membrane (0.9 cm^2^).

**Quantification analysis**

RT-PCR was performed in 3 non-, 1 ng/ml and 10 ng/ml IL-13 treated models from 3 different donors (N=9). We analyzed the mRNA expression of forkhead box protein J1 (FOXJ1) which is a [transcription factor](https://en.wikipedia.org/wiki/Transcription_factor) involved in ciliogenesis, MUC5AC which is expressed by mucus producing cells, Club (Clara) Cell Protein (CC10/CC16) (club cell protein) which is produced by nonciliated club cells and Keratin 5 (KRT5) which is expressed primarily in basal cells [3]. Fibroblasts were gently scraped from the model and the total mRNA of PBEC was isolated by PureLink® RNA Mini Kit (Life technologies, Paisley, UK). First-strand cDNA was synthesized from 0.2 ng of total RNA using High-Capacity RNA-to-cDNA™ Kit (Life technologies, Paisley, UK). The Fast SYBR^®^ Green Master Mix (Life technologies, Paisley, UK) was used to perform the RT-PCR with respective primers. The primers were designed by software Primer 3 and purchased from CyberGene (Stockholm, Sweden). Beta actin was adopted as an internal control gene. 50 ng cDNA was used in each 20 μl PCR reaction volume to identify the products of interest. Data were analyzed using 7500 Software v.2.0.1 and transformed using the ΔCt method. The results were then calculated as 2^-ΔΔCt^ (ΔΔCt = [ΔCt (gene of interest, airlifted model in different conditions)] - [ΔCt (gene of interest, model without airlifted)] with ΔCt = Ct (gene of interest) - Ct (beta actin)), and expressed as fold change (2^-ΔΔCt^ of 1or 10 ng/ml IL-13 treated model/ 2^-ΔΔCt^ of non-IL-13 treated model).

**References**

1. von Scheele I, Larsson K, Palmberg L. Budesonide enhances Toll-like receptor 2 expression in activated bronchial epithelial cells. Inhal Toxicol. 2010;22(6):493-9. Epub 2010/04/15. doi: 10.3109/08958370903521216. PubMed PMID: 20388003.

2. Strandberg K, Palmberg L, Larsson K. Effect of budesonide and formoterol on IL-6 and IL-8 release from primary bronchial epithelial cells. J Asthma. 2008;45(3):201-3. Epub 2008/04/17. doi: 10.1080/02770900801890372792211122 [pii]. PubMed PMID: 18415826.

3. Schamberger AC, Staab-Weijnitz CA, Mise-Racek N, Eickelberg O. Cigarette smoke alters primary human bronchial epithelial cell differentiation at the air-liquid interface. Sci Rep-Uk. 2015;5. doi: Artn 816310.1038/Srep08163. PubMed PMID: ISI:000348670200004.
